# The genome sequence of the Whirlpool Ramshorn snail, *Anisus vortex *(Linnaeus, 1758)

**DOI:** 10.12688/wellcomeopenres.19836.1

**Published:** 2023-08-16

**Authors:** Sue Skipp, Jonathan Ablett, Natural History Museum Genome Acquisition Lab, Darwin Tree of Life Barcoding collective, Wellcome Sanger Institute Tree of Life programme, Wellcome Sanger Institute Scientific Operations: DNA Pipelines collective, Tree of Life Core Informatics collective, Darwin Tree of Life Consortium

**Affiliations:** 1 Environment Agency, Rochester, UK; 2 Natural History Museum, London, England, UK

**Keywords:** Anisus vortex, Whirlpool Ramshorn snail, genome sequence, chromosomal, Hygrophila, Planorbidae, Lymnaeoidea

## Abstract

We present a genome assembly from an individual *Anisus vortex *(the Whirlpool Ramshorn snail; Mollusca; Gastropoda; Hygrophila; Lymnaeoidea; Planorbidae). The genome sequence is 869.5 megabases in span. Most of the assembly is scaffolded into 18 chromosomal pseudomolecules. The mitochondrial genome has also been assembled and is 13.57 kilobases in length.

## Species taxonomy

Eukaryota; Opisthokonta; Metazoa; Eumetazoa; Bilateria; Protostomia; Spiralia; Lophotrochozoa; Mollusca; Gastropoda; Heterobranchia; Euthyneura; Panpulmonata; Hygrophila; Lymnaeoidea; Planorbidae; *Anisus*; *Anisus vortex* (Linnaeus, 1758) (NCBI:txid271030).

## Background

*Anisus vortex*, better known as the Whirlpool Ramshorn snail, is distributed across Europe and Western Asiatic, reaching up to Finland in the Arctic circle ( Kerney, 1999). It is commonly found in lowland areas in standing or slow-moving rivers, canals, lakes and drainage ditches however is not tolerant of habitats prone to desiccation ( Kerney, 1999; Welter-Schultes, 2012). *Anisus vortex* is listed as a species of least concern in the current IUCN Red List data ( Moorkens *et al.*, 2011).

In the UK *Anisus vortex* is abundant in southern, eastern and central England as well as central Ireland. It is absent or only found in localised areas in the north of England and Scotland ( Kerney, 1999; Rowson *et al.*, 2021). Some older records from the most easterly and northern Scottish reports may be misidentifications for *Anisus leucostoma* ( Kerney, 1999).

**Figure 1.**
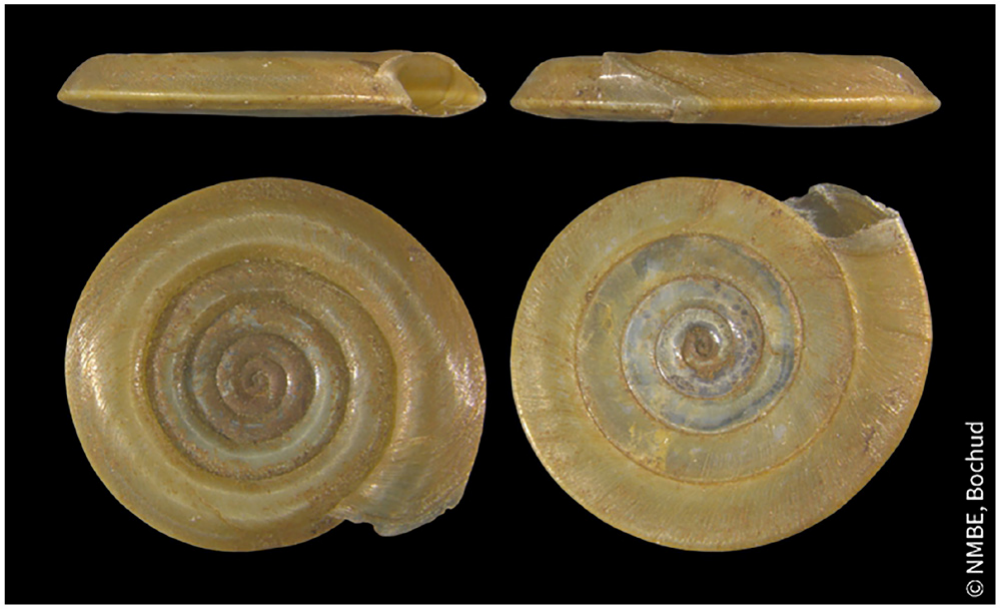
Photograph of *Anisus vortex* shells by Estée Bochud (CC-BY-NC-SA).

The shell is 7–10 mm in diameter, 1–2 mm in thickness and nearly opaque, yellow/brown in colour and is thin, smooth and glossy consisting of 6 to 7 whorls ( Figure 1). The sharp keel is runs along the upper edge of the shell, whilst the underside is more rounded giving the aperture a more rhomboid like shape ( Glöer, 2002; Rowson *et al.*, 2021). The live animal is commonly dark purple-grey with white or colourless tentacles ( Rowson *et al.*, 2021). Juveniles are proportionally thicker in size but can be easily recognised by the strongly displaced keel ( Rowson *et al.*, 2021).

*Anisus vortex* is easily distinguished from other genera within the family by the sharp keel and angled aperture ( Rowson *et al.*, 2021). *Anisus vortex* can be easily confused with *A. vorticulus*, which is much rarer with more specific habitat requirements. These species can be separated by the more central keel in *A. vorticulus* and the more rhomboid profile of the shell in *A. vortex* ( Kerney, 1999) or by dissection of the genital anatomy ( Rowson *et al.*, 2021).

Eggs are up to 0.5 mm in diameter and are laid in capsules, ovoid in shape and around 4 mm long, each containing 10 to 12 eggs ( Rowson *et al.*, 2021; Welter-Schultes, 2012).

Along with other planorbid species, *Anisus vortex* has been shown to be an intermediate parasite host to several species of vertebrate parasites including *Alaria alata*, which has been shown to infect domestic dogs as well as foxes ( Portier *et al.*, 2012).

The genome of *Anisus vortex* was sequenced as part of the Darwin Tree of Life Project, a collaborative effort to sequence all named eukaryotic species in the Atlantic Archipelago of Britain and Ireland. Here we present a chromosomally complete genome sequence for *Anisus vortex*, based on one specimen from Pocklington Canal, York, UK.

## Genome sequence report

The genome was sequenced from one *Anisus vortex* collected from Pocklington Canal, York, UK (53.89, –0.85). A total of 43-fold coverage in Pacific Biosciences single-molecule HiFi long reads was generated. Primary assembly contigs were scaffolded with chromosome conformation Hi-C data. Manual assembly curation corrected 46 missing joins or misjoins and removed 15 haplotypic duplications, reducing the assembly length by 0.7%, and decreasing the scaffold N50 by 4.99%.

**Figure 2.**
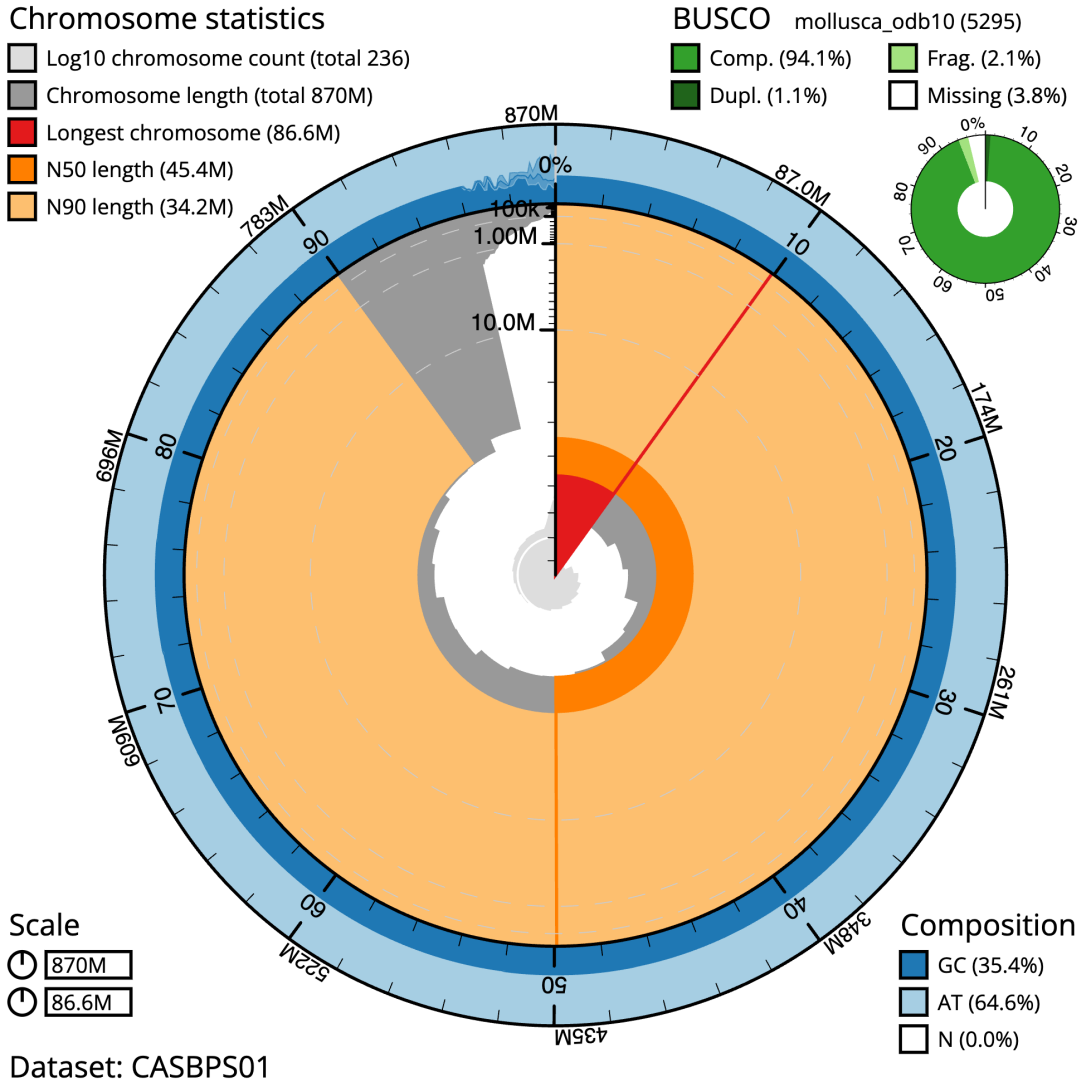
Genome assembly of *Anisus vortex*, xgAniVort1.1: metrics. The BlobToolKit Snailplot shows N50 metrics and BUSCO gene completeness. The main plot is divided into 1,000 size-ordered bins around the circumference with each bin representing 0.1% of the 869,544,677 bp assembly. The distribution of scaffold lengths is shown in dark grey with the plot radius scaled to the longest scaffold present in the assembly (86,562,697 bp, shown in red). Orange and pale-orange arcs show the N50 and N90 scaffold lengths (45,417,137 and 34,151,741 bp), respectively. The pale grey spiral shows the cumulative scaffold count on a log scale with white scale lines showing successive orders of magnitude. The blue and pale-blue area around the outside of the plot shows the distribution of GC, AT and N percentages in the same bins as the inner plot. A summary of complete, fragmented, duplicated and missing BUSCO genes in the mollusca_odb10 set is shown in the top right. An interactive version of this figure is available at https://blobtoolkit.genomehubs.org/view/Anisus%20vortex/dataset/CASBPS01/snail.

The final assembly has a total length of 869.5 Mb in 235 sequence scaffolds with a scaffold N50 of 45.4 Mb ( Table 1). Most (96.27%) of the assembly sequence was assigned to 18 chromosomal-level scaffolds. Chromosome-scale scaffolds confirmed by the Hi-C data are named in order of size ( Figure 2– Figure 5; Table 2). While not fully phased, the assembly deposited is of one haplotype. Contigs corresponding to the second haplotype have also been deposited. The mitochondrial genome was also assembled and can be found as a contig within the multifasta file of the genome submission.

**Table 1.** Genome data for *Anisus vortex*, xgAniVort1.1.

Project accession data		
Assembly identifier	xgAniVort1.1	
Species	*Anisus vortex*	
Specimen	xgAniVort1	
NCBI taxonomy ID	271030	
BioProject	PRJEB59083	
BioSample ID	SAMEA7520804	
Isolate information	xgAniVort1: whole organism (DNA sequencing) xgAniVort3: whole organism (Hi-C scaffolding)	
Assembly metrics *		*Benchmark*
Consensus quality (QV)	59.7	*≥ 50*
*k*-mer completeness	100%	*≥ 95%*
BUSCO **	C:94.1%[S:93.0%,D:1.1%], F:2.1%,M:3.8%,n:5,295	*C ≥ 95%*
Percentage of assembly mapped to chromosomes	96.27%	*≥ 95%*
Sex chromosomes	-	*localised homologous pairs*
Organelles	Mitochondrial genome assembled	*complete single alleles*
Raw data accessions		
PacificBiosciences SEQUEL II	ERR10798433, ERR10802389	
Hi-C Illumina	ERR10802456	
Genome assembly		
Assembly accession	GCA_949126835.1	
*Accession of alternate haplotype*	GCA_949126815.1	
Span (Mb)	869.5	
Number of contigs	655	
Contig N50 length (Mb)	3.5	
Number of scaffolds	235	
Scaffold N50 length (Mb)	45.4	
Longest scaffold (Mb)	86.6	

* Assembly metric benchmarks are adapted from column VGP-2020 of “Table 1: Proposed standards and metrics for defining genome assembly quality” from ( Rhie *et al.*, 2021). ** BUSCO scores based on the mollusca_odb10 BUSCO set using v5.3.2. C = complete [S = single copy, D = duplicated], F = fragmented, M = missing, n = number of orthologues in comparison. A full set of BUSCO scores is available at https://blobtoolkit.genomehubs.org/view/Anisus%20vortex/dataset/CASBPS01/busco.

**Figure 3.**
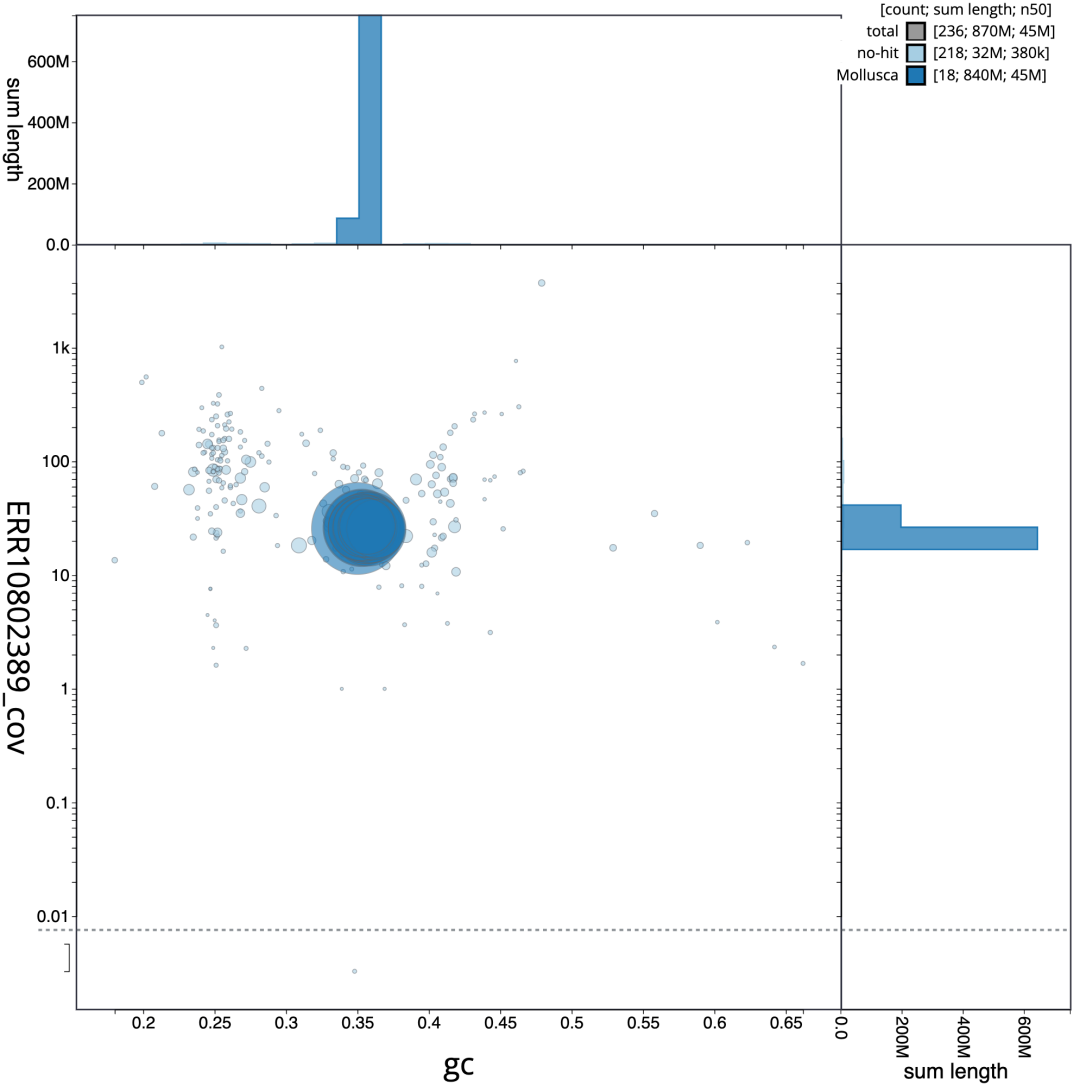
Genome assembly of *Anisus vortex*, xgAniVort1.1: BlobToolKit GC-coverage plot. Scaffolds are coloured by phylum. Circles are sized in proportion to scaffold length. Histograms show the distribution of scaffold length sum along each axis. An interactive version of this figure is available at https://blobtoolkit.genomehubs.org/view/Anisus%20vortex/dataset/CASBPS01/blob.

**Figure 4.**
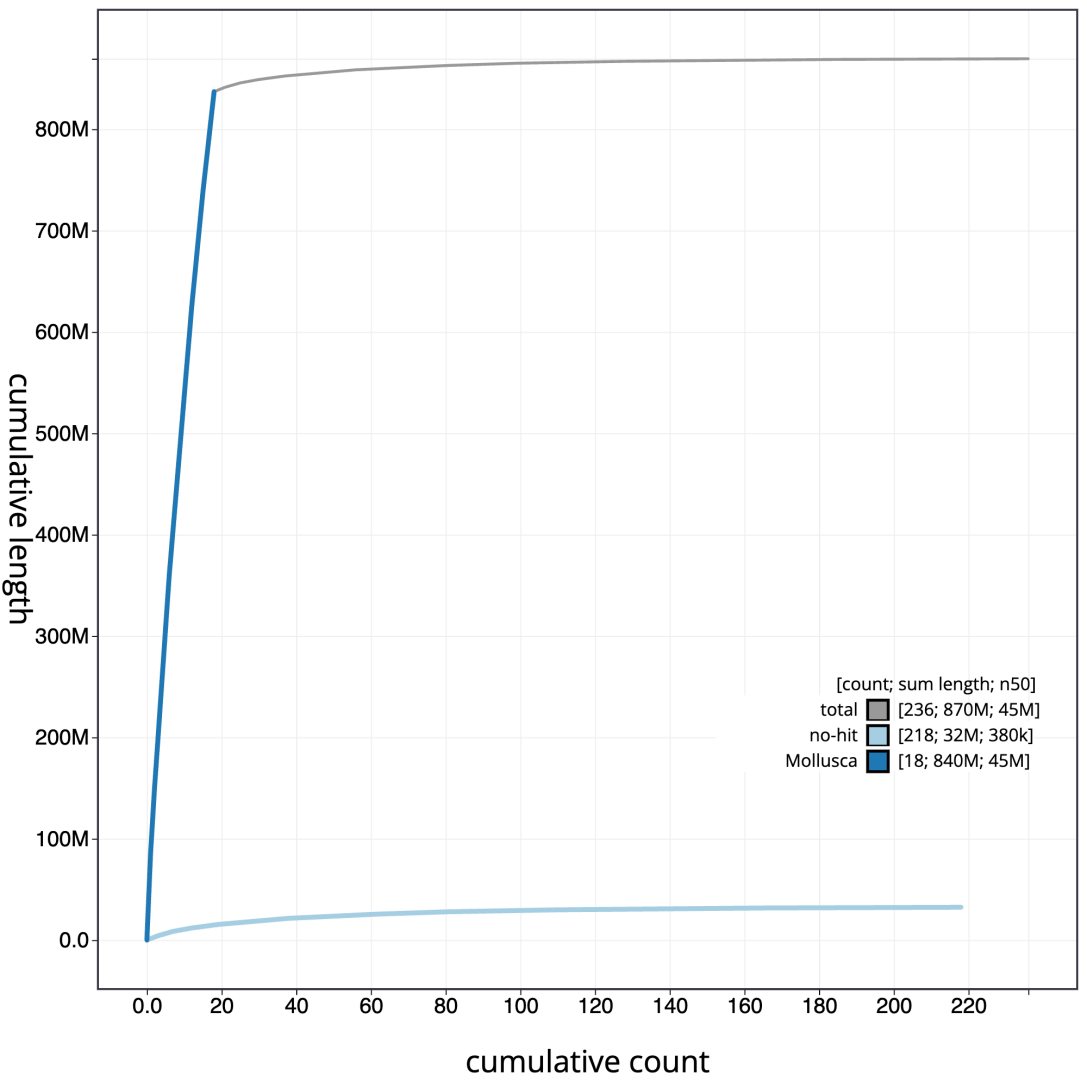
Genome assembly of *Anisus vortex*, xgAniVort1.1: BlobToolKit cumulative sequence plot. The grey line shows cumulative length for all scaffolds. Coloured lines show cumulative lengths of scaffolds assigned to each phylum using the buscogenes taxrule. An interactive version of this figure is available at https://blobtoolkit.genomehubs.org/view/Anisus%20vortex/dataset/CASBPS01/cumulative.

**Figure 5.**
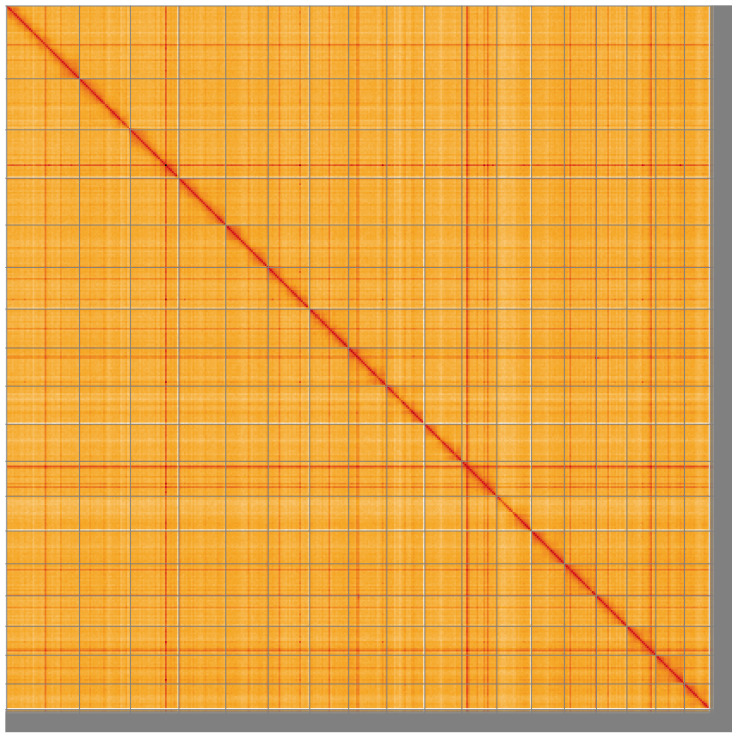
Genome assembly of *Anisus vortex*, xgAniVort1.1: Hi-C contact map of the xgAniVort1.1 assembly, visualised using HiGlass. Chromosomes are shown in order of size from left to right and top to bottom. An interactive version of this figure may be viewed at https://genome-note-higlass.tol.sanger.ac.uk/l/?d=Sbo4O9XrQoWcCW4SkeAH4A.

**Table 2.** Chromosomal pseudomolecules in the genome assembly of *Anisus vortex*, xgAniVort1.

INSDC accession	Chromosome	Length (Mb)	GC%
OX421492.1	1	86.56	35.0
OX421493.1	2	60.78	35.5
OX421494.1	3	57.64	35.5
OX421495.1	4	55.67	36.0
OX421496.1	5	50.57	35.5
OX421497.1	6	49.49	35.5
OX421498.1	7	46.29	35.5
OX421499.1	8	45.42	36.0
OX421500.1	9	45.27	35.5
OX421501.1	10	44.06	35.5
OX421502.1	11	41.62	35.5
OX421503.1	12	41.36	35.0
OX421504.1	13	39.0	36.0
OX421505.1	14	38.11	35.5
OX421506.1	15	36.27	35.5
OX421507.1	16	34.44	35.5
OX421508.1	17	34.15	36.5
OX421509.1	18	30.45	35.5
OX421510.1	MT	0.01	25.0

The estimated Quality Value (QV) of the final assembly is 59.7 with *k*-mer completeness of 100%, and the assembly has a BUSCO v5.3.2 completeness of 94.1% (single = 93.0%, duplicated = 1.1%), using the mollusca_odb10 reference set ( *n* = 5,295).

Metadata for specimens, spectral estimates, sequencing runs, contaminants and pre-curation assembly statistics can be found at https://links.tol.sanger.ac.uk/species/271030.

## Methods

### Sample acquisition and nucleic acid extraction

An *Anisus vortex* (specimen ID NHMUK014360733, individual xgAniVort1) was collected from Pocklington Canal, UK (latitude 53.89, longitude –0.85) on 2019-03-19 using a kicknet. The specimen was collected and identified by Sue Skipp (Environment Agency), and was then snap-frozen in a dry shipper. 

The xgAniVort1 sample was prepared at the Tree of Life laboratory, Wellcome Sanger Institute (WSI). The sample was weighed and dissected on dry ice. Whole organism tissue was disrupted using a Nippi Powermasher fitted with a BioMasher pestle. DNA was extracted at the Wellcome Sanger Institute (WSI) Scientific Operations core using the Qiagen MagAttract HMW DNA kit, according to the manufacturer’s instructions.

### Sequencing

Pacific Biosciences HiFi circular consensus DNA sequencing libraries were constructed according to the manufacturers’ instructions. DNA sequencing was performed by the Scientific Operations core at the WSI on Pacific Biosciences SEQUEL II and SEQUEL IIe instruments. Hi-C data were also generated from whole organism tissue of xgAniVort3 using the Arima2 kit and sequenced on the Illumina NovaSeq 6000 instrument.

### Genome assembly, curation and evaluation

Assembly was carried out with Hifiasm ( Cheng *et al.*, 2021) and haplotypic duplication was identified and removed with purge_dups ( Guan *et al.*, 2020). The assembly was then scaffolded with Hi-C data ( Rao *et al.*, 2014) using YaHS ( Zhou *et al.*, 2023). The assembly was checked for contamination and corrected as described previously ( Howe *et al.*, 2021). Manual curation was performed using HiGlass ( Kerpedjiev *et al.*, 2018) and Pretext ( Harry, 2022). The mitochondrial genome was assembled using MitoHiFi ( Uliano-Silva *et al.*, 2023), which runs MitoFinder ( Allio *et al.*, 2020) or MITOS ( Bernt *et al.*, 2013) and uses these annotations to select the final mitochondrial contig and to ensure the general quality of the sequence.

A Hi-C map for the final assembly was produced using bwa-mem2 ( Vasimuddin *et al.*, 2019) in the Cooler file format ( Abdennur & Mirny, 2020). To assess the assembly metrics, the *k*-mer completeness and QV consensus quality values were calculated in Merqury ( Rhie *et al.*, 2020). This work was done using Nextflow ( Di Tommaso *et al.*, 2017) DSL2 pipelines “sanger-tol/readmapping” ( Surana *et al.*, 2023a) and “sanger-tol/genomenote” ( Surana *et al.*, 2023b). The genome was analysed within the BlobToolKit environment ( Challis *et al.*, 2020) and BUSCO scores ( Manni *et al.*, 2021; Simão *et al.*, 2015) were calculated.

Table 3 contains a list of relevant software tool versions and sources.

**Table 3.** Software tools: versions and sources.

Software tool	Version	Source
BlobToolKit	4.1.5	https://github.com/blobtoolkit/blobtoolkit
BUSCO	5.3.2	https://gitlab.com/ezlab/busco
Hifiasm	0.16.1-r375	https://github.com/chhylp123/hifiasm
HiGlass	1.11.6	https://github.com/higlass/higlass
Merqury	MerquryFK	https://github.com/thegenemyers/MERQURY.FK
MitoHiFi	2	https://github.com/marcelauliano/MitoHiFi
PretextView	0.2	https://github.com/wtsi-hpag/PretextView
purge_dups	1.2.3	https://github.com/dfguan/purge_dups
sanger-tol/genomenote	v1.0	https://github.com/sanger-tol/genomenote
sanger-tol/readmapping	1.1.0	https://github.com/sanger-tol/readmapping/tree/1.1.0
YaHS	1.2a	https://github.com/c-zhou/yahs

### Wellcome Sanger Institute – Legal and Governance

The materials that have contributed to this genome note have been supplied by a Darwin Tree of Life Partner. The submission of materials by a Darwin Tree of Life Partner is subject to the **‘Darwin Tree of Life Project Sampling Code of Practice’**, which can be found in full on the Darwin Tree of Life website here. By agreeing with and signing up to the Sampling Code of Practice, the Darwin Tree of Life Partner agrees they will meet the legal and ethical requirements and standards set out within this document in respect of all samples acquired for, and supplied to, the Darwin Tree of Life Project. 

Further, the Wellcome Sanger Institute employs a process whereby due diligence is carried out proportionate to the nature of the materials themselves, and the circumstances under which they have been/are to be collected and provided for use. The purpose of this is to address and mitigate any potential legal and/or ethical implications of receipt and use of the materials as part of the research project, and to ensure that in doing so we align with best practice wherever possible. The overarching areas of consideration are:

•   Ethical review of provenance and sourcing of the material

•   Legality of collection, transfer and use (national and international) 

Each transfer of samples is further undertaken according to a Research Collaboration Agreement or Material Transfer Agreement entered into by the Darwin Tree of Life Partner, Genome Research Limited (operating as the Wellcome Sanger Institute), and in some circumstances other Darwin Tree of Life collaborators.

## Data Availability

European Nucleotide Archive: *Anisus vortex* (whirlpool ramshorn); Accession number PRJEB59083; https://identifiers.org/ena.embl/PRJEB59083. ( Wellcome Sanger Institute, 2023) The genome sequence is released openly for reuse. The *Anisus vortex* genome sequencing initiative is part of the Darwin Tree of Life (DToL) project. All raw sequence data and the assembly have been deposited in INSDC databases. The genome will be annotated using available RNA-Seq data and presented through the Ensembl pipeline at the European Bioinformatics Institute. Raw data and assembly accession identifiers are reported in Table 1.

## Author information

Members of the Natural History Museum Genome Acquisition Lab are listed here: https://doi.org/10.5281/zenodo.4790042.

Members of the Darwin Tree of Life Barcoding collective are listed here: https://doi.org/10.5281/zenodo.4893703.

Members of the Wellcome Sanger Institute Tree of Life programme are listed here: https://doi.org/10.5281/zenodo.4783585.

Members of Wellcome Sanger Institute Scientific Operations: DNA Pipelines collective are listed here: https://doi.org/10.5281/zenodo.4790455.

Members of the Tree of Life Core Informatics collective are listed here: https://doi.org/10.5281/zenodo.5013541.

Members of the Darwin Tree of Life Consortium are listed here: https://doi.org/10.5281/zenodo.4783558.
