# Supplementary material for: The genome sequence of the Whirlpool Ramshorn snail, Anisus vortex (Linnaeus, 1758)
Source: Wellcome Open Res. 2023 Aug 16;8:344. [Version 1] doi: 10.12688/wellcomeopenres.19836.1 (PMC10477736; doi:10.12688/wellcomeopenres.19836.1)
Supplement: Peer review history

# Peer review: Reviewer response for version 1

**DOI:** 10.21956/wellcomeopenres.21968.r65476

**Published:** 2023-09-04

**Authors:** David Ferrier

**Affiliations:** 1 University of St Andrews, St Andrews, Scotland, UK

The rationale is clear. Besides the need for sequencing this mollusc as part of the Darwin Tree of Life project, there is the added interest of this animal being an intermediate host for a parasitic trematode that can infect mammals such as dogs and cats, but also potentially humans.

 The various protocols are certainly appropriate and the work is of a high technical standard.

 The materials and methods descriptions meet accepted standards for these data notes, and the datasets such as the assemblies of the two haplotypes are accessible and useable.

 There are a couple of minor typos for correction.

1. Typo in Background, third paragraph: delete ‘is’ from “The sharp keel is runs along”.
2. Genome sequence report section, second paragraph, line 8: insert a space to read, “haplotype have”.

Are sufficient details of methods and materials provided to allow replication by others?

Yes

Is the rationale for creating the dataset(s) clearly described?

Yes

Are the datasets clearly presented in a useable and accessible format?

Yes

Are the protocols appropriate and is the work technically sound?

Yes

Reviewer Expertise:

NA

I confirm that I have read this submission and believe that I have an appropriate level of expertise to confirm that it is of an acceptable scientific standard.

# Peer review: Reviewer response for version 1

**DOI:** 10.21956/wellcomeopenres.21968.r65479

**Published:** 2023-08-21

**Authors:** Jerome H L Hui

**Affiliations:** 1 The Chinese University of Hong Kong, Hong Kong, China

Skipp and colleagues report the genome sequence of freshwater ramshorn snail *Anisus vortex *(Linnaeus 1758). This species can be found in most places in Britain. Molecular data of this species are mainly confined to COI and ribosomal sequences deposited to the NCBI database. Since this species has also been found to be intermediate host of parasitic diseases in dogs and foxes, this new genome resource is important and will be very useful for further studies, such as understanding and prevention of the transmission of diseases, as well as evolutionary and genomics questions related to molluscs more widely.

 This genome resource is excellent from the summary statistics, with high BUSCO numbers, high sequence continuity (scaffold N50), and majority of sequences contained on the 18 pseudochromosomes (plus mitochondrion). To sum up, this is a valuable contribution.

Are sufficient details of methods and materials provided to allow replication by others?

Yes

Is the rationale for creating the dataset(s) clearly described?

Yes

Are the datasets clearly presented in a useable and accessible format?

Yes

Are the protocols appropriate and is the work technically sound?

Yes

Reviewer Expertise:

Genomics, evolution, invertebrates

I confirm that I have read this submission and believe that I have an appropriate level of expertise to confirm that it is of an acceptable scientific standard.
